# Health policy considerations for combining exercise prescription into noncommunicable diseases treatment: a narrative literature review

**DOI:** 10.3389/fpubh.2023.1219676

**Published:** 2023-10-02

**Authors:** Dan Tao, Roger Awan-Scully, Garrett I. Ash, Yaodong Gu, Zhong Pei, Yang Gao, Alistair Cole, Rashmi Supriya, Yan Sun, Rui Xu, Julien S. Baker

**Affiliations:** ^1^Faculty of Sports Science, Ningbo University, Ningbo, China; ^2^Research Academy of Medicine Combining Sports, Ningbo No.2 Hospital, Ningbo, China; ^3^Department of Government and International Studies, Hong Kong Baptist University, Kowloon Tong, Hong Kong SAR, China; ^4^Section of General Internal Medicine, Yale School of Medicine, Yale University, New Haven, CT, United States; ^5^Center for Pain, Research, Informatics, Medical Comorbidities and Education Center (PRIME), VA Connecticut Healthcare System, West Haven, CT, United States; ^6^Department of Neurology, The First Affiliated Hospital, Sun Yat-Sen University, Guangzhou, China; ^7^Department of Sports, Physical Education and Health, Hong Kong Baptist University, Kowloon Tong, Hong Kong SAR, China; ^8^School of Sports and Health, Nanjing Sport Institute, Nanjing, China; ^9^Centre for Health and Exercise Science Research, Hong Kong Baptist University, Kowloon Tong, Hong Kong SAR, China

**Keywords:** exercise prescription, health policy, noncommunicable diseases, medical provision, health policy triangle framework

## Abstract

**Objectives:**

In this review, we aim to highlight the evidence base for the benefits of exercise in relation to the treatment of noncommunicable diseases (NCDs), draw on the Health Triangular Policy Framework to outline the principal facilitators and barriers for implementing exercise in health policy, and make concrete suggestions for action.

**Methods:**

Literature review and framework analysis were conducted to deal with the research questions.

**Results:**

Exercise prescription is a safe solution for noncommunicable diseases prevention and treatment that enables physicians to provide and instruct patients how to apply exercise as an important aspect of disease treatment and management. Combining exercise prescription within routine care, in inpatient and outpatient settings, will improve patients’ life quality and fitness levels.

**Conclusion:**

Inserting exercise prescription into the healthcare system would improve population health status and healthy lifestyles. The suggestions outlined in this study need combined efforts from the medical profession, governments, and policymakers to facilitate practice into reality in the healthcare arena.

## 1. Introduction

The 2011 United Nations Declaration on Noncommunicable Diseases (NCDs) recognized the importance of NCDs as a global health issue, particularly for low- and middle-income countries (1). NCDs are highly prevalent, costly, and are responsible for more than 74% of deaths worldwide, totaling 41 million people each year (2). In all but 20 countries, prior to the Covid-19 pandemic individuals were at a greater risk of premature death from NCDs than from all communicable diseases, maternal problems, perinatal issues, and poor nutrition combined (3). An estimated 17 million people die globally from NCDs before the age of 70, and 86% of these recorded premature deaths occur in low- and middle-income countries (4, 5). The four main groups of NCDs are cardiovascular diseases, cancers, chronic respiratory diseases, and diabetes. These diseases are responsible for over 80% of premature NCD deaths. Disease onset has several underlying modifiable risk factors, including using tobacco, the use of excessive alcohol, sedentary behavior, and unhealthy diets (6). The patient suffering and family economic burden resulting from NCDs is associated with profound negative consequences for families, health care systems and countries (7). The United Nations has estimated that the burden of chronic disease cumulative cost to the global economy may reach $47 trillion by 2030 if current trends do not change (8). NCDs have been included in the Sustainable Development Goal (SDG) Target 3.4. This target has been established to reduce premature mortality by one-third by 2030, through improved NCDs treatment and prevention, in addition to promoting mental health and well-being (3, 9).

The beneficial impact of exercise on health has been well documented since the 5^th^ century BC; this relationship has been investigated, defined and reinforced by many years of scientific research. The evidence indicates a strong and consistent positive association between exercise participation and health status (10–14). Many systematic reviews and meta-analyses have revealed the comprehensive benefits that can be obtained from physical exercise (15–19). Exercise as a validated methodology has endured the test of time and can help individuals develop healthy lifestyles (20). Exercise is also a proven applied intervention for addressing illness and improving health and wellness (21). For example, exercise has been used in both the prevention and treatment of many chronic conditions such as pulmonary disease, heart disease, obesity, and diabetes (22, 23). Each year, hundreds of billions of dollars are invested in healthcare provision for NCDs; exercise interventions can often provide similar and/or greater health benefits as those provided by pharmaceutical interventions, without most of the associated expenses or the problematic side-effects (24, 25). In some instances, exercise can complement and influence traditional medicine treatments while enhancing the medicinal positive effect (26). The World Health Organization provides general exercise guidelines and recommendations for different age groups and specific populations. These groups include postpartum and pregnant women, individuals that have chronic conditions, and the disabled. The guidelines outline in brief the quantity of physical activity that is required for health benefits (27). However, the guidance is not targeted for individual NCD patients and cannot be used to prescribe individualized specific exercise programmes.

Exercise prescription (EP), based on the findings of previous studies, can be defined as: written and structured instruction by supervised/medical staff that establishes and uses supported exercise programmes that are clearly defined and contain elements of exercise advice stating exercise type, intensity, duration and frequency. Prescribing exercise should also include the patient’s health, exercise ability and cardio-pulmonary function based on medical examination and treatment outcomes (24, 25). Exercise prescription can be implemented by clinical exercise physiologist and qualified exercise professional physicians in a variety of settings (28). However, an interview study completed at the national level in the United States revealed that only 32% of patients received advice about exercise or the benefits of continuing to be physically active during visits with a physician (29). Physician counselling and exercise prescription referrals may be helpful for reducing morbidity and mortality rates from NCDs (30). Unfortunately, most physicians, who are traditionally trained (pharmaceutically or medically) to manage noncommunicable disease have not been extensively trained in exercise prescription at their associated medical schools or healthcare institutions (30).

Enhancing primary health care using exercise prescription is a low-risk, cost-effective approach to maximize health benefits at population levels. For this to be achieved there is a requirement for medical systems to create the necessary infrastructure and environment to ensure that supervised exercise can be prescribed as medicine. Exercise prescription is an additional healthcare provision that enables physicians to support their patients to engage in exercise as part of their disease prevention and treatment (22). Realizing these benefits to health care needs to be facilitated by large-scale investment from governments, non-government organizations, and the private sectors (2). Healthcare systems around the world are highly disparate, and vary according to the level of development in a particular country, and in local and national public health service provision. For example, inequality in health services coverage is common across South Asia, and the service does not achieve the key global target of at least 80% of essential health care service coverage (2). A 2020 World Health Organization survey revealed that the ongoing COVID-19 pandemic was disrupting NCD services in 122 of 159 countries; the combination of COVID-19 and NCDs has had devastating consequences for many patients’ access to health systems (5). There needs to be a stronger political commitment, the development of institutional and human capacity, and creative sustainable solutions to finance health systems that offer the widespread provision of exercise prescription within medical services at affordable rates (2).

Most intervention and review studies designed around physical activity and exercise therapy are terminated at the efficacy trial stage, without transfer as best practices for clinical provision systems and public health policy-making processes (16, 18, 31–34). As the available evidence indicates that exercise is effective as a treatment for NCDs, exercise treatment should be sufficiently recognized as a stimulating and beneficial policy change in the healthcare and wellness agenda. In the previous two decades, numerous health policy studies have focused on how to manage and improve treatment performance and outcomes (35). However, public health policy changes can also be implemented to provide a strategy to create better conditions for population health.

Based on the narrative provided above, this article aims to provide evidence, and outline a feasible plan for consideration, to implement exercise as medicine for future NCDs medical treatment provision. This article has been divided into three main sections. First, the article outlines the evidence base underpinning exercise as a medicine for NCDs. Second, the article discusses the health policy-making process, drawing on the Health Triangular Policy Framework. Third, the article presents specific suggestions for exercise prescription in the medical provision system.

## 2. Methods

### 2.1. Literature review

A structured comprehensive search strategy was conducted using PubMed, Google Scholar, Embase, Web of Science, Scopus and ProQuest to identify publications in English using search terms in the titles and abstracts (search strategies were adjusted to fit different databased setting): ‘exercise prescription’, ‘exercise treatment’, ‘health policy’, ‘medical system’, ‘clinical provision’, ‘healthcare’, ‘inpatient’, ‘outpatient’, ‘noncommunicable diseases’, ‘cardiovascular diseases’, ‘cancer’, ‘respiratory diseases’, ‘diabetes’. The search process was focused on original studies and reviews articles (in peer-reviewed journals) over the last 15 years. Further related articles were identified from reference lists of retrieved articles; and authority data (e.g., WHO) that were also considered. A total of 76 articles were finally included and the literature search was completed on November 16, 2022.

### 2.2. Framework analysis

**Figure 1:**
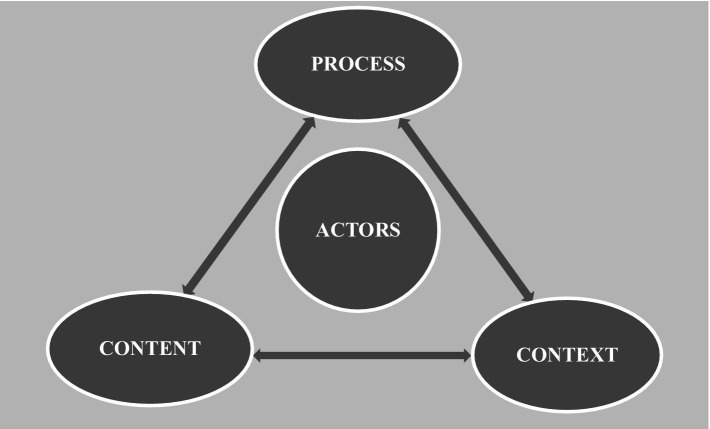
Health policy triangle framework (HPF) model.

Our health policy-making analysis below is based around the Health Triangular Policy Framework (HPF) (36). The HPF was developed in 1994 by Wall and Gilson, and has been used in a retrospective or prospective manner to analyze many health-related policy issues (37). The framework highlights how policy implementation is influenced by the elements of policy *Content*, *Context*, and *Process*, and also emphasizes the influence of *Actors* on these three elements in the policy-making process (38). In the HPF, *Content* includes policy objectives, operational policies, legislation, regulations, and guidelines. *Context* refers to systemic factors: social, economic, political, cultural, and other environmental conditions. *Process* refers to the way in which policies are initiated, developed or formulated, negotiated, communicated, implemented, and evaluated. *Actors* refer to influential individuals (such as senior policy- and decision-makers), groups, and organizations (see Figure 1) (36). Studies have used this framework to analyze health policy issues in contexts as diverse as Kenya (39), Cambodia (40), and the Eastern Mediterranean regions (37).

## 3. Results

### 3.1. Exercise as a treatment for NCDs

Physical exercise is increasingly recognized as of importance in the primary medical care of at least 35 chronic diseases. Exercise not only reduces the risk of developing new chronic NCDs, but also decreases the progression of many existing chronic noncommunicable conditions while improving quality of life (41, 42). Specifically, the significance of exercise intervention as the first-line treatment for several chronic NCDs has been investigated extensively during the past two decades (26). Exercise may induce modifications in gene expression, and affect cardiovascular risk, musculoskeletal function, pulmonary function, hyperinsulinemia, sensitivity to insulin, oxygen consumption, fasting plasma/blood glucose intolerance, body fats, cholesterol, blood pressure, dosage of antidiabetic medications, immunity, sleep quality, self-satisfaction, and consequently general health and fitness. Furthermore, exercise can also improve aerobic capacity, and the mental health of patients (7, 17, 33, 34, 43).

Not everything is known, of course, and there remains important work to be done to explore the different types and dosage of exercise that are suitable for exercise prescription following specific diagnoses and conditions for NCD patient populations. A 2008 American College of Sports Medicine (ACSM) report noted, for example, that Moderate to Vigorous Physical Activity (MVPA) reduced breast and colon cancer risk. In 2018, the ACSM report was expanded to include more types of cancers whose risk was reduced by MVPA. These included endometrial, esophageal, kidney, lung, stomach, and bladder cancers (7, 41). Engagement in regular exercise activity is not only feasible and safe for cancer patients but also improves the tolerance for treatment, facilitates recovery, reduces the length of hospital stays, slows progression, and reduces risks associated with recurrence, readmission, and mortality (44).

### 3.2. Exercise intervention guidelines and education for NCD patients

The ‘Health Care Providers Action Guide’ was developed by the ACSM. It outlines six basic steps for working with health care providers and patients to facilitate and manage medical referrals, including assessment of the physical activity levels of patients, determination of patients’ readiness to change, prescription of exercise for patients, provision of patients’ physical activity referrals, and promotion of exercise in clinics (45). Several resources have also been developed, including exercise implementation tools, handouts, and patient reports. Examples such as physical activity algorithms with an initial assessment by a nurse or clinician, link into exercise prescription and patient education, concluding with suggested readings to encourage patients to change behavior from inactive to active (46). Adaptation of the physical activity algorithm into clinical practice allows for an opportunity to increase patients’ participation in their own health outcomes using a prescribed exercise routine. Similar forms of guidance have been developed by ACSM and other organizations: examples include ACSM guidelines on using exercise for cancer survivors (47); the Enhanced Recovery After Surgery (ERAS) programme (48); and the Pulmonary rehabilitation exercise prescription in chronic obstructive pulmonary disease guidelines etc. (49).

Exercise education and consultation are further relevant factors for patients with NCDs. The identification of patient intervention capacity and suitability can be achieved by considering the various contraindications and constraints, preferences, barriers and facilitators for exercise, and the potential benefits relevant to them, whether those be physiological, psychosocial or economic (50). Further steps, aims and objectives of exercise need to be prioritized according to the most valuable outcome for the patients (e.g., symptom management, improving mood, minimizing a decline in cardiorespiratory fitness, reversing loss of muscle mass, survival etc.). Therefore, it is essential to provide the patients with information relating to the exercise components that are necessary for achieving specific goals. It is worth noting here that some may prioritize long-term exercise benefits over short-term goals, physiological over psychosocial or functional benefits, and that the goals and priorities for many will likely change over time (50). The flexibility of exercise education and consultation are particularly important when patient exercise preferences fail to align with achieving their desired goals (50). Physical exercise education and consultation are facilitators for NCDs patients to participate in exercise programmes. The skills and knowledge developed by patient education and exercise guidelines may provide confidence and competence in minimizing adverse reactions to related individual medical situations, such as the fear of hypoglycemia. Additionally, health professional training and improving community engagement is also essential in exercise prescription support following discharge, based on the physician’s guidance (51).

### 3.3. Multidisciplinary team care

The scope of exercise prescription practice is dynamic and can be responsive to the needs of patients and society. The ideal condition is for health services to provide adequate recognition and support for interdisciplinary intervention through proper legislation and policies (52). Unstructured referral from physicians deprives patients of medical services such as diagnosis, health promotion, and complication prevention (53). The most significant considerations for implying multidisciplinary team care are cost and convenience (54). In primary care, physicians, nurses, physiotherapists, and other health professionals working together in a medical services team is an effective strategy to achieve multidisciplinary health service goals. Furthermore, at the primary health care level, organizing people-centered integrated health services that are safe for patients and of assured quality with effective referral networks between basic health care and hospital care is essential (53, 55, 56). Multidisciplinary practice can be aided by standardized procedures, policies, and regulations. Furthermore, the organizational structure of service delivery and clinical responsibilities suggests that it can be responsive to the needs of patients and communities rather than practitioner-led delivery (53).

Health clubs and personal trainers are traditional outlets for the promotion of physical activity and exercise regimens, and physicians are not depending on these partnerships to promote physical activity to their patients in a sufficient manner (54). Not only is inter-hospital collaboration required, collaboration between physicians and community practitioners is an important link for patients with NCDs after they leave the clinic or are discharged from the hospital (54). Fostering multisectoral public and commercial partnerships that bring together government and society to promote health policies is vital to address concerns such as financing, accessibility, efficiency, and the quality of health care (57).

### 3.4. Barriers to exercise prescription implementation

From a patient perspective, common reasons for non-adherence to exercise prescription include physical and psychosocial factors such as patient missed sessions, dropouts because of wellness problems, hospital readmissions, disease progressions, adverse skeletal events, or a paucity of interest, motivation, knowledge and/or confidence (24, 44, 51, 58). From an institutional and physician perspective, major barriers for service integration are related to limited funding, lack of a detailed implementation plan, limited availability of suitable programmes, and low organizational buy-in (lack of public funding and resources). In addition, the physicians’ characteristics of lacking exercise science educational backgrounds, related knowledge, and specific professional qualifications have also been noted as detrimental (24, 32, 44, 51, 58). Furthermore, in real work situations the lack of time for counselling, workload pressures, and extra work concerns, would all disrupt clinic efficiency and have been identified as related issues contributing to barriers for providing exercise prescription to patients (58). Some accessibility barriers related to location, cost and exercise schedules coinciding with treatment days have also been identified. In addition, political and economic context arrangements, such as minimal structured reimbursement policies; and barriers related to collaboration and leadership with stakeholder and other actor groups in social context are also issues that cannot be ignored (58).

### 3.5. Related examples

Among the documented international examples of effective uses of exercise in the prevention and treatment of NCDs are the following three initiatives:

- Singapore has adapted a systematic approach for integrating exercise as medicine into the disease and management pathway. This resulted in the design of an Exercise as Medicine template, and progressively incorporated the Physical Activity Vital Sign (PAVS) structure into electronic case notes in numerous departments at Changi General Hospital. The mainstay of Exercise is Medicine (funded in 2007 by the American College of Sports Medicine) in the Singapore development is to train physicians in the prescription of exercise (22).
- Another example is the implementation of tailored exercise interventions for women following breast cancer diagnosis in Brisbane, Australia. A supervised exercise intervention led to significant improvements in fitness parameters and significant decreases in breast cancer health care utilization (32) This provided a cost-effective treatment compared to usual care with eight-year follow-up (59).
- A further example comes from the Jewish General Hospital, Montreal, Quebec. During the operation of a Rehabilitation and Exercise Oncology model of care (ActivOnco), patients were encouraged to participate in exercise based on individual goals and preferences in a group environment or individual setting (60). Most of the referrals (71%) came from members of the multidisciplinary team, including nutritionists, nurse coordinators, social workers, and treating oncologists (60). The programme benefited from easy access to medical records, and interaction with medical teams providing constant updates on the treatment and medical status for patients and patients’ families (50).

### 3.6. Health policy analysis

Health policy design comprises a set of decisions, plans and actions taken within a society to achieve a specific healthcare goal, or a set of actions taken by an agency or organization at the national, regional or local levels of government to promote public health (36). Based on the evidence discussed above we can reasonably claim that NCDs patients would be greatly advantaged by a healthcare system shift in conceptualization toward an exercise health promotion initiative, including re-orientation of the general practice environment and collaboration with exercise professionals in inpatient and outpatient provision. At a societal level, healthcare support includes developing practical infrastructure, equipment base and peer networks; and improvements in the confidence of patients for exercise interventions, making them more receptive to exercise prescription. The following table outlines the health policy-making analysis for implementation of exercise prescription in regard to NCDs (See Table 1).

**Table. 1:** The health policy-making analysis for implementation of exercise prescription into the noncommunicable diseases medical provision based on the health triangular policy framework.

Items	Details
Actors	*National and regional governments* that develop the policy and local governments that deliver and implement the policy *The hospitals* where exercise prescription is specially used *Medical schools and universities* that provide resources for physicians who are qualified to prescribe exercise *Business organizations* that provide the resources for exercise tests and exercise practice equipment *Community healthcare providers* that ensure follow-up to exercise prescription *Researchers* in the field that provide valid references to guide both healthcare providers and patients The *patients* with NCDs
Content	The *provision of medical healthcare* for NCDs is modified to include exercise prescription within routine care for the patient as the main content A *physician’s professional qualification* in exercise prescription is a basic requirement Evidence-based *exercise guidelines and consulting guidance principles* that can be tailored for the individual patient into scientific professional exercise prescription *Implementation* of the prescription, including instruction and guidance for individual patients *Follow-up monitoring* of how patients implement the prescription after they leave the clinic or are discharged from the hospital, and the impact on their health
Context	The *hospital or clinic setting* – the inpatient or outpatient environment – where patients and medical professionals interact The *medical training system* where expertise in exercise prescription can be acquired The *domestic, local, broader societal environments and home-based exercise programme* for all patients receiving exercise prescription *Political, economic, cultural, social, national, regional, local, and international factors* that can affect or influence policy development, and shape the context for health policy-making
Process	*Assess* possible exercise intervention options based upon a theoretical foundation from scientific sources of evidence and information *Analyze* the patients’ health level or medical needs, and the alignment of actual activities implemented with the proposed policies *Break down the boundaries* between traditional health exercise science and exercise as a medicine treatment *Develop* detailed health policy choices for providing inpatient and outpatient exercise interventions, then choose the most cost-effective options *Implement* policy in the inpatient and outpatient settings of both primary and secondary healthcare *Ensure* that relevant legislation and government policies are consistent with and supportive of the implementation of exercise prescription *Evaluate* the policy effect

## 4. Discussion

### 4.1. Applying a formal exercise prescription curriculum in the physician training system

There is evidence that providing effective exercise guidance for patients can help increase physical activity levels and decrease disease rates, thereby reducing the financial burden on governments (28). However, exercise prescription or physical activity advice for patients with NCDs are not included in the academic curriculum of most medical professionals (30, 61, 62). A previous survey outlined a lack of formal undergraduate medical education knowledge related to the medical benefits of exercise and physical activity in 1975. Subsequent surveys since 1975 have demonstrated little improvement (30). For example, most US medical students do not have the competence, skills, or confidence to counsel patients on exercise prescription following graduation (30). It is essential that newly qualified physicians are aware of the role that exercise as medicine can play in treating and preventing disease. A more recent study reported that exercise guidelines and exercise prescription education programmes provide the tools and knowledge to assess properly patients’ activity levels and offer individualized recommendations, while also increasing practitioner confidence to engage in exercise prescription (28).

Physicians may lack knowledge and confidence to prescribe exercise as medicine because of the paucity of detailed instruction in medical schools (63). Training and education for physicians on the benefits of different modes of exercise prescription, and how to structure exercise intervention to each patient’s needs, can empower them with confidence and knowledge (28). Conceptualization and implementation of an exercise prescription medical education course is needed to provide health care staff with the ability to assess, counsel, and refer patients for physical activity and exercise. This development is crucial to prevent and treat chronic noncommunicable diseases and is required urgently (22). Exercise prescription training needs to be integrated into most of phases of medical education, and can be facilitated in medical schools, residency programmes, credentialing processes and continuing education requirements (30). The development and implementation of professional standards must include performance and ethics, standards of conduct, and core clinical skills. They need to be established under a standards development framework in which all aspects are evidence-based, benchmarked against international standards such as those of the American College of Sports Medicine, and consistent with the needs of patients and practitioners (61). Assessment of the quality of education and training programmes is also required, to ensure they give clinical exercise physiology graduates the skills and knowledge to practice safely and competently. A university curricula programme accreditation framework should be developed based on current national health service provision models (61). There may also be a need to include specialized mandatory courses and conduct an evaluation of exercise prescription within the physicians’ final examination for the degree.

There is also a requirement to develop physicians’ communication skills to enable them to guide patients in the behavioral change scenarios of exercise adaptation and maintenance in daily life. Furthermore, physicians need to be encouraged to engage with community physical activity/exercise resources, which is consistent with the evolution of patient-centered care (30). When medical students graduate, they should be able to demonstrate proficiency in the prescription of exercise and assessment; and should also be knowledgeable about the principles of exercise counselling and behavioral strategies related to patients’ personal health (30). Additionally, developing collaborations between medical schools/universities and healthcare institutions not only provides more exercise medicine practical information, but also provides internship opportunities.

There is a programme available in the US called Exercise is Medicine Greenville (EIMG). This is the first partnership engaging a medical school, a health care system, and a community organization combining resources collectively to educate physicians on the clinical benefits of exercise (22). Several universities have now successfully incorporated sports and exercise medicine into their medical curriculum in places as diverse as Colombia, South Carolina, the United Kingdom, and Iran. These curricula have included a focus on exercise medicine and how to successfully prescribe exercise for patients (30, 64–67). This extensive system needs further development, consolidation, expansion, and exploration as a model of best practice.

### 4.2. Guarantee adequate exercise medicine resources

The installation of an exercise prescription facility within existing NCDs medical care provision settings may require slight infrastructural adjustment to ensure the availability of suitable spaces, and any necessary equipment, to provide instruction in different forms of exercise. There will also be a need for the hiring of experienced exercise science specialists, and medical staff who possess exercise prescription skills. A concerted effort is required to identify and develop the correct implementation strategies to stimulate a cultural shift and debate in the host organization (44).

Exercise testing is necessary when health/fitness and clinical exercise professionals are concerned about individuals’ risk, or when they require additional information to design exercise prescription (68). An 18-year cross-sectional study provided data on the safety of clinical Chinese population-based exercise testing, and was expressed as the number of adverse events per 10,000 tests (using 95% confidence intervals). These results suggested that clinical exercise testing was safe and that the low incidence of adverse events recorded might be due to the overall changes in clinical practice over time (69). In real practice, the clinical exercise test would need to meet three important aspects: address patients’ needs, be time efficient, and be cost-effective. NCDs patients should be screened using a health risk appraisal questionnaire or a self-reported medical history (including patients and family health history, comorbidities, additional chronic diseases and related treatments, and physical activity and exercise history). An example would be the modified American Heart Association/ACSM Health/Fitness Facility Preparticipation Screening Questionnaire (41) for the presence of risk factors for various cardiovascular, pulmonary, renal, and metabolic diseases as well as other conditions such as pregnancy and orthopedic injury that require special attention when developing exercise prescription. Further medical evaluation measures, including blood pressure, heart rate and some anthropometric indicators should also be conducted to determine any health issues that contribute to risks of morbidity and/or mortality. Physical activity assessment needs to be incorporated into standard medical examination routines during clinical visiting and as part of the treatment plan (22). This recommendation should be considered not only for patients at high risk of exercise-related complications; regular exercise testing should be recommended for all patients prior to participating in a light- to moderate-intensity exercise programme (68). Evidence-based, specific, and valid measures of physical status would aid physicians in examination and discussion of exercise prescription with patients (70).

Technology-based support, such as mobile-technology, wearable devices, texts, and emails, improves compliance among patients and has been validated previously (31, 71–73). Suggestions to design and develop special software/applications to enhance exercise prescription adherence are gaining momentum. Digital support tools can be used for the purpose of including information such as the patient’s medication history, illness, and physical fitness levels; follow-ups using remote monitoring after outpatient’s service or discharge should be attractive to many healthcare systems and providers. Using these methodologies, patients can live at home and by using multiple intelligent devices communicate with the physician to access supervision and instructions. This communication helps with follow-up over the next weeks or months to monitor compliance with exercise prescription and provide encouragement. This method can also expand the opportunities for increased accuracy and acceptance of exercise intervention. Remote consultation is also featured by consistency and regularity, low cost and acceptability to the patient’s family. In addition to the digital health system mentioned previously, the workflows for exercise prescription will need to be compatible with existing workflows and working procedures to limit burdens and increase efficiency (24).

Community exercise resources are another important component for effective exercise continuation in compliance with physician’s instructions following discharge. Health providers and physicians will often wish to refer patients to community resources to integrate exercise into their daily lives. These include the use of self-directed resources, such as public sports facilities, bike-sharing programmes, and the use of local parks (22). Aligning community and healthcare partners to provide a clinic-to-community model may be beneficial for implementing exercise as a core prevention strategy for assisting patients who are at risk for NCDs, to regress, reverse, and minimise the progression of disease (74).

### 4.3. Effective evaluation for exercise prescription implementation

For general practitioners and fitness related personnel, focusing on the theoretical study and practical operation of exercise interventions for NCDs needs to be re-evaluated. The potential way to ensure the quality of exercise prescription is the development of an exercise prescription credential to recognize qualified and certified fitness professionals who work with patients referred from health care providers (22). This includes medical practice, health care knowledge, NCDs prevention and treatment, exercise rehabilitation, and scientific exercise understanding. Follow-up evaluation after the implementation of the policy should not be ignored, and investigations and surveys should be conducted to further determine the effectiveness of exercise prescription implementation, as well as patients’ confidence levels and intervention effects.

### 4.4. Including exercise prescription into medical insurance programmes

**Figure. 2:**
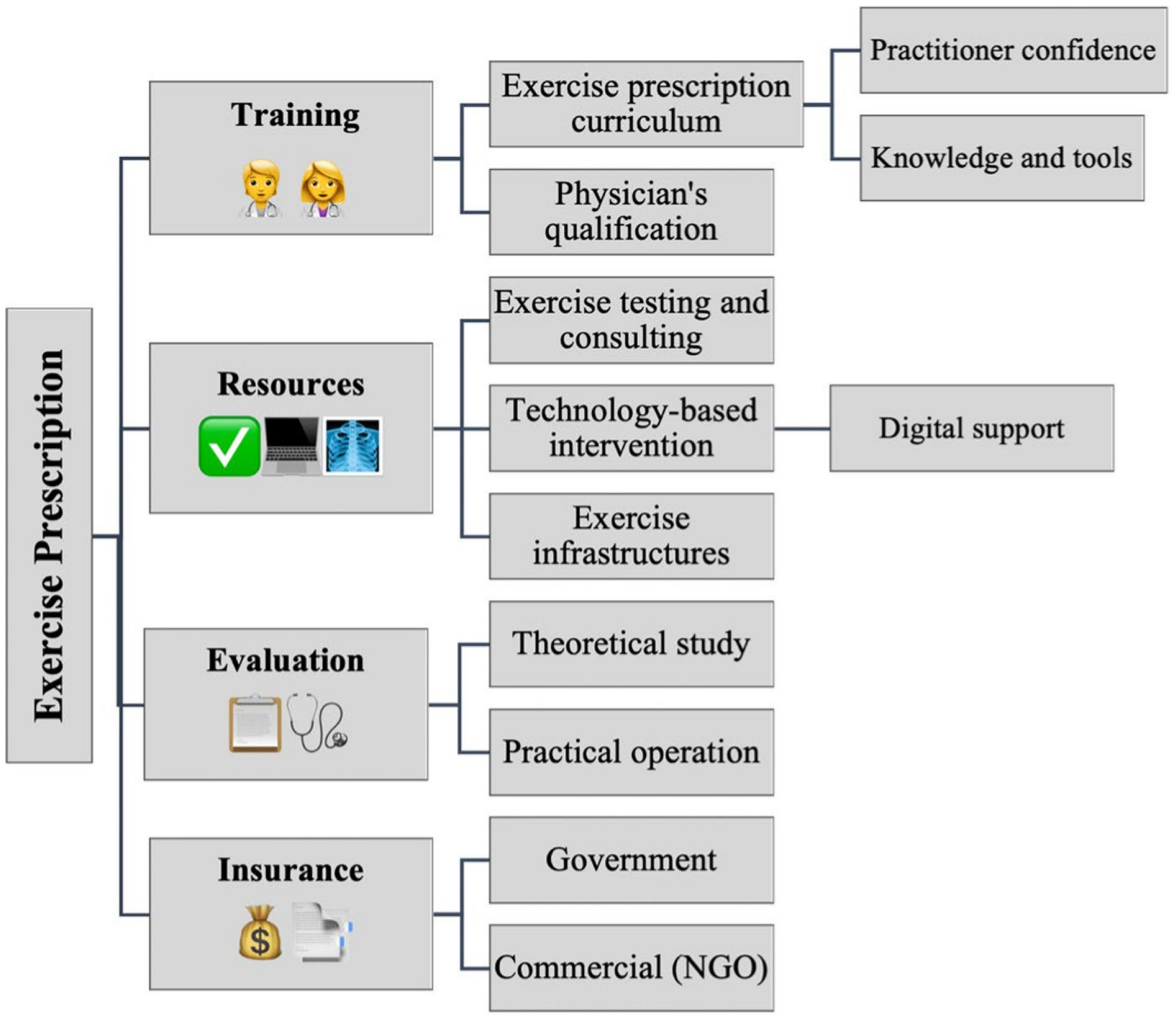
Map of exercise prescription implementation.

Achieving universal health coverage (UHC) is a sub-target of the Sustainable Development Goals (SDGs) (36). The acceptance and coverage of government universal medical insurance and commercial insurers to include exercise prescription in the claim for reimbursement is the cornerstone of long-term development of exercise prescription. Medical insurance system support is essential for physicians and patients to adhere to exercise prescription. If medical insurance policies generally provide more reimbursement for inpatient care, this incentivizes patients to use hospital resources for even minor health conditions, and therefore inhibits healthcare treatment from primary clinics (75). The government’s healthcare policy could support the treatment of most NCDs in outpatient settings. This would provide savings in medical resources and medical expenses and improve the health level of the entire population in the long term. In addition, this provision would increase the treatment capacities of related hospitals and healthcare establishments. For example, outpatient medical body checks/tests related to patient symptoms and physical exercise ability, should be part of every physician’s exercise prescription, and follow-up exercise medical assistance should be reimbursed as usual. The implementation of exercise prescription in clinical provision would reduce the expenditure of drugs, intravenous infusion therapy and inpatient care. On the other hand, it also indirectly reduces the expenses of inpatient medical reimbursement and provides a virtuous healthy cycle. Nevertheless, medical insurance policies should set a limited coverage for exercise prescription in primary health care/clinic provision, by means of setting a maximum limit for total reimbursement, to avoid over-use of medical services. Figure 2 provides a general outline of the suggestions of implementation of exercise prescription.

## 5. Conclusion

Based on the available evidence, exercise is a powerful intervention to prevent and manage NCDs, improve patients’ life quality and mitigate the effects of chronic diseases, and lower mortality rates. Exercise prescription is a non-pharmaceutical health intervention and should be promoted in combination with traditional medical support. Individual patients will be at different stages of readiness to participate in exercise, and each will present with different unique health and environmental challenges, so the individual prescription of exercise recommendation is necessary and beneficial. This review summaries the evidence and suggestions for implementing exercise prescription into the NCDs medical healthcare provision. The information provided here also identifies barriers that need to be overcome for success in implementation of the health policy-making process. There is a paucity of studies documenting the process of exercise as medicine in different and varied healthcare settings. This gap creates difficulties for providers, in the selection and choice of appropriate strategies at organizational levels that recognize the factors enabling the adaption and development of new working methods. Healthcare professionals also need to be aware that prescribing exercise as medicine in one particular setting may not be applicable and beneficial in the same way when implemented elsewhere. Medical practitioners need to select and modify appropriate and useful strategies at each organizational level, recognizing the combination of factors that enable development and adoption of new working practices.

Inserting exercise prescription into the healthcare system would improve population health status and healthy lifestyles. We have attempted to link the literature to the structure of the specific policy-making process, bridging the gap from theory to practical implication suggestions. Following the scientific policy-making process and suggestions, we need to keep the general alignment between policy and practice to ensure long-term success, effective implementation, and delivery. The suggestions outlined in this study need combined efforts from the medical profession, governments, and policymakers to facilitate practice into reality in the healthcare arena.

## Author contributions

DT performed writing-original draft, conceptualization, and data interpretation. DT and JSB performed the literature searches and contributed to the screening process and selection of included studies. DT, RA-S, GIA, and JSB performed writing-review and editing, verification. ZP, YDG, YG, AC, RS, YS, and RX provided critical feedback on the protocol. YDG performed project administration. All authors contributed to the article and approved the submitted version.
